# Glycemic responses to maize flour stiff porridges prepared using local recipes in Malawi

**DOI:** 10.1002/fsn3.293

**Published:** 2015-10-07

**Authors:** Vincent Mlotha, Agnes Mbachi Mwangwela, William Kasapila, Edwin W.P. Siyame, Kingsley Masamba

**Affiliations:** ^1^ Faculty of Food and Human SciencesLilongwe University of Agriculture and Natural Resources (LUANAR)P.O. Box 219LilongweMalawi

**Keywords:** Consumer behavior, diabetes mellitus, diet, fermentation, *ugali*

## Abstract

Glycemic index is defined as the incremental area under the blood glucose response curve of a 50 g carbohydrate portion of a test food expressed as a percent of the response to the same amount of carbohydrate from a standard food taken by the same subject. This study investigated glycemic index of maize stiff porridges consumed as staple food in Malawi and a large majority of other countries in sub‐Saharan Africa to identify areas for improvement in consumer diets. Stiff porridges were prepared using flour from whole maize, maize grits, and fermented maize grits. The porridges were served to 11 healthy volunteers for 3 weeks, with two serving sessions a week. Glucose was served as a reference food during weekly serving sessions. Results from descriptive analysis revealed that glycemic responses varied across subjects and porridge types. Porridge prepared from fermented maize grits had moderate glycemic index of 65.49 and was comparable in nutrient composition and sensory characteristics with the other test porridges. Glycemic indices of the porridges prepared from whole maize flour and grits were high at 94.06 and 109.64, respectively, attributed to the effect of traditional maize flour processing, preparation, and cooking methods used. The study also calculated glyaemic load of the porridges and drew recommendations to inform diet planning and modifications for healthy and diabetic individuals.

## Introduction

There is a growing interest in glycemic index (GI) of carbohydrates‐rich foods to help consumers make healthy food choices within specific food groups. Glycemic index is defined as the incremental area under the blood glucose response curve of a 50 g carbohydrate portion of a test food expressed as a percent of the response to the same amount of carbohydrate from a standard food taken by the same subject (FAO and WHO, 1998). It is used to classify carbohydrate foods based on their blood glucose raising potential. Low‐GI foods are those that are digested and absorbed slowly, resulting in low fluctuations in blood sugar levels (Brand‐Miller et al. 2014). Good examples of such foods are whole grain cereals, whole kernel bread, beans, and fruits. High‐GI foods, by virtue of their rapid digestion and absorption, produce marked fluctuations in blood sugar levels and they include white bread and highly processed grains, cereals, and potatoes (Brand‐Miller et al. 2014).

Various authors have studied in vitro digestion models that mimic in vivo situation to examine diets and their effect on postprandial glycemia (Kelly et al. 2004). Many interventions and epidemiological studies have also investigated the short‐term biological and health effects of foods, meals, and diets of varying GI to understand implications on health (Meyer et al. 2000; Foster‐Powell et al. 2002; Benton et al. 2003; Rizkalla et al. 2004; Fatema et al. 2013). At present, there is global research‐based evidence that reductions in daily glycemic load (GL) may lead to a reduced risk for developing noncommunicable diseases (NCDs), such as type 2 diabetes, cancer, and coronary heart disease.

In 1998, Food and Agriculture Organization (FAO) and World Health Organization (WHO) recommended low‐GI diets (GI of 0–55) as a viable way to prevent and address the burden of NCDs. In several countries (Australia, France, Sweden, Canada, and South Africa), the use of the GI concept has been integrated in dietary guidelines given by health professionals, and an increasing number of food companies market low‐GI products (Brouns et al. 2005). In line with these developments, a large number of academic and commercial laboratories have undertaken measurements of glycemic indices of food for both research and commercial application purposes (Brouns et al. 2005).

Glycemic indices of food have also been examined and reported in various studies conducted elsewhere in Africa (Foster‐Powell et al. 2002; Mahgoub et al. 2013). Unfortunately, reported GI values vary widely across studies and cannot be used to guide diets for the region. More research is needed in a wider variety of countries to get population specific data necessary for guiding nutritionists, healthcare practitioners, and consumers.

In sub‐Saharan Africa, it is estimated that 12.1 million people are living with diabetes and this figure is projected to increase to 23.9 million by 2030 (Msyamboza et al. 2014). Malawi is one of the countries affected by the NCD epidemic in the region. For example, results from a 2009 WHO STEPS survey showed that in every 100 Malawian adults six have diabetes, 33 have hypertension and five are obese (Msyamboza et al. 2014). A large majority of patients in the country suffer from type 2 diabetes. Type 2 diabetes occurs when blood glucose levels are higher (>108 mg/dL) than normal (68–108 mg/dL), and can be prevented through health food choices, physical activity, and weigh loss. Diabetes type 1 is caused by genetic factors, such as shortage of insulin and decreased ability to use insulin, a hormone that allows glucose (sugar) to enter cells and be converted to energy. Blood sugar levels rise and lead to the disease in this regard.

In tandem with the global strategy to address NCDs, the Malawi government through the Ministry of Health has included dietary management of cardiovascular diseases and diabetes among priorities of essential health package for 2011–2016 (Government of Malawi, 2012). Unfortunately, glycemic index data for local carbohydrate‐rich foods are nonexistent and nutrition labeling, which also help consumers make informed food choices, is voluntary and not well understood, when available on food packaging, by the majority of consumers.

**Figure 1:**
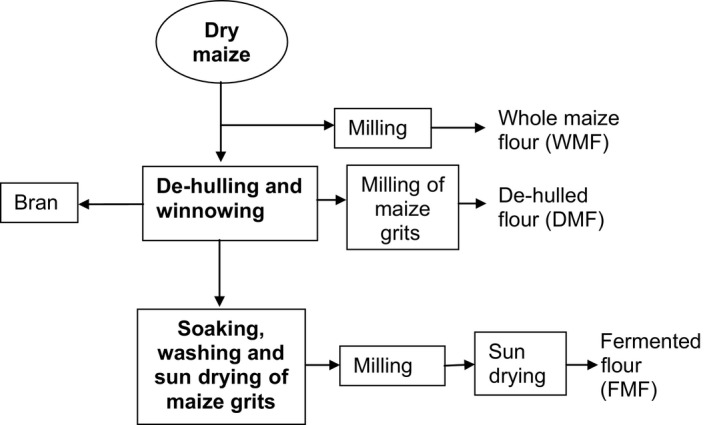
Processing of maize into different flours. Source: Matumba et al. (2009); Mpulula (2013).

*Nsima* is a local dish made from either maize flour and eaten widely in Malawi and across Africa where it has different local names, including *Ugali* in eastern Africa and *fufu* in western Africa. It is usually eaten together with side dishes locally known as relish—legumes, meat, fish, and vegetables—and various chilli and tomato sauces. Figure 1 shows stages in preparation of different flours from maize. The aim of this study was to determine the glycemic index of maize stiff porridges consumed locally as staple food in Malawi to help consumers make informed choices about low‐GI foods.

## Materials and Methods

### Ethical approval for the study

The study protocol was reviewed and approved by the National Health Sciences Research Committee (NHSRC) under the approval number NHSRC 1130, having been approved by the Department of Food Science and Technology at Lilongwe University of Agriculture and Natural Resources. Medical examinations of the subjects, finger pricking, and blood glucose determination were done by three registered nurses at the University clinic. All subjects provided formal consent before participation in the study. On the other hand, researchers guaranteed confidentiality of information obtained and abided by professional ethical conduct such as neutrality, respect for respondent's dignity, culture, and data verification.

### Recruitment of research subjects

An announcement was made on the notice boards of the University for healthy volunteers, students, and staff of both sexes, to participate in this study. Thirteen subjects responded to the call and were screened for eligibility. Screening involved diagnosis of insulin sensitivity, glucose tolerance status, and diabetes to minimize outlier glycemic response to test foods. Weight and height were taken to determine levels of overweight and obesity. Subjects were also asked about their past and current medical history as part of medical examination for the study. A total of 12 subjects were deemed eligible and recruited. Participants were also informed about study objectives and commitment required from them—to avoid heavy meals, alcohol, smoking, and vigorous physical activity a day before and on the morning of the test. Other study protocols such as participant's availability at 8:00 h once a week for a period of 2 months and the requirement to consume study samples and have finger‐prick tests were communicated. Researchers reiterated that participation is voluntary and that one could withdraw from the study at any time after having given consent to participate. One subject withdrew and the study was conducted on 11 participants.

### Test foods for the study

The test food for this study was *nsima* prepared traditionally from three flours known locally as *mgaiwa*,* gramil* and white cornmeal (*woyera*). Thick porridges were prepared from maize flour and water. Porridges were stirred for 5 min to create a stiff paste by adding more flour. Portioning was done using a wooden spoon into serving sizes containing 50 g carbohydrate determined using food composition database and an analytical balance. These portions were 399.91 g, 292.71 g, and 252.77 g for *nsima* prepared from the above‐mentioned flours, respectively. Two GI testing sessions took place every week for a period of 3 weeks. At each session of the week, subjects consumed a particular type of *nsima* and glucose, the reference food (Table 1). Nutrient composition of flours used was determined using Association of Official Analytical Chemists (AOAC) methods.

**Table 1:** GI testing sessions for the study

Week	Day	Food material
1	Tuesday, 16 April 2013	Glucose
	Friday, 19 April 2013	Whole maize flour thick porridge
2	Monday, 22 April 2013	Glucose
	Thursday, 25 April 2013	Dehulled, degermed maize flour thick porridge
3	Monday, 29 April 2013	Glucose
	Thursday, 2 May 2013	Fermented dehulled and degermed maize grits thick porridge

### Preparation of standard food

Glucose powder (Royale, batch number 1001) was purchased from a local pharmacy and used as a standard food for this study. Fifty grams of glucose was measured using an analytical balance (Sartorius, L01201S, serial number 50511449) and diluted into 250 mL of tap water.

### Measurement of Glycemic Index

Many methods can be used to calculate GI of food. This study used incremental area under the curve (incremental AUC) method as recommended by FAO and WHO (1998). This method has been used for most calculations of GI (Foster‐Powell et al. 2002; Brouns et al. 2005). Measured portions of test foods containing 50 g of available carbohydrate were served to subjects at 8:00 h, breakfast time. Capillary finger‐prick blood samples were taken at 0 (fasting), 15, 30, 45, 60, 90, and 120 min after starting to eat the test meal using a safety lancet and glucose stick. Blood glucose was determined using glucometers (Betachek® G5, Blood glucose monitoring system, National Diagnostic Products, Sydney, NSW, Australia). These blood glucose values were used to calculate the incremental area under the curve (iAUC), a reflection of the total rise in blood glucose levels after eating the test food. Steps for calculation of glycemic index using iAUA are given in Table 2.

**Table 2:** Steps in GI Calculation using iAUA

1. Recruitment of healthy individuals
2. Defining the amount of the test food containing 50 g of glycemic carbohydrates
3. Determining the standard food to be used (white bread or glucose)
4. Preparation and serving of test/standard food
5. GI testing sessions and collection of capillary blood samples over a 2‐hour period for analysis
6. Calculating individual GI ratings by dividing blood glucose responses for test food by reference food
7. Calculating the GI value for the test food as an average GI value for the 10 people
8. Classification of food

### Data analysis

All analyses were done using the SPSS software for Windows (version 16) (SPSS Inc., 2006, Chicago, IL). The incremental areas under the curve (iAUC) were calculated by the standardized criteria (FAO and WHO, 1998), ignoring any area below the baseline. The average iAUC for the three glucose tests was used as the reference value, and each subject's individual GI for each food was calculated. Glycemic values were calculated by dividing the iAUC for the test foods by the iAUC for the reference food and multiplying by 100. The average of the glycemic ratings from all eleven subjects was recorded as the GI for that food. Glycemic load of each food was calculated by multiplying the amount of available carbohydrate in a typical serving of the food and the GI of that food divided by 100 (Table 2).

## Results

### Characteristics of the study subjects

The study engaged 12 health subjects, seven male and five female, with an average age of 24.5 years, mean BMI of 21.76 kg/m^2^ and normal average fasting blood glucose of 3.92 mmol/L. The majority of them spent most of their time resting and doing light physical exercises (Table 3).

**Table 3:** Socio‐demographic data for the study volunteers

	Mean ± SD
Socio‐demographic characteristic	
Age (Years)	23.48 ± 3.52
Weight (kg)	59.06 ± 7.38
Height (cm)	164.89 ± 5.38
BMI (kg/m^2^)	21.76 ± 3.06
Fasting blood glucose (mmol/L)	3.92 ± 0.53
Physical activity (hours)	
Resting (includes sleeping)	12.09 ± 5.19
Light exercise	8.18 ± 4.96
Moderate exercise	3.45 ± 2.38
Heavy exercise	0.27 ± 0.65

Values are means and standard deviation (SD) of 11 subjects.

### Composition of stiff porridges

Table 4 shows the composition of the test foods for this study. Maize flours used to prepare these foods were processed differently that contributed to variations in composition of the porridges. Stiff porridge made from whole maize flour contained more nutrients than the other two porridges, with exception of carbohydrate. For example, a 399.91 g serving portion of whole maize flour stiff porridge that provided 50 g glycemic carbohydrate contained 11.36 g of protein, 28.27 g of fat, and 2.3 g of crude fiber, figures that were higher than those for the other two porridges (Table 4). FAO (1992) states that maize portion that remains after removal of the hulls and germ through processing are chiefly composed of starch (Fig. 1).

**Table 4:** Proximate composition of thick porridges prepared from different maize flours (g/100 g)

Parameter	Whole maize flour	Dehulled, degermed maize flour	Maize flour made from fermented dehulled and degermed maize grits	*P*‐value
Moisture	76.54 ± 0.33^a^	78.54 ± 0.02^b^	76.46 ± 0.71^a^	0.002
Ash	0.48 ± 0.03^b^	0.26 ± 0.08^a^	0.22 ± 0.08^a^	0.007
Crude protein	2.84 ± 0.44^b^	1.63 ± 0.46^a^	1.64 ± 0.42^a^	<0.001
Crude fat	7.07 ± 0.75^b^	2.26 ± 0.17^a^	1.71 ± 0.96^a^	<0.001
Crude fiber	0.58 ± 0.08^b^	0.23 ± 0.08^a^	0.19 ± 0.05^a^	0.009
Carbohydrate	13.08	17.31	19.97	

Values are means of three determinations and means with different superscripts in the same row are significantly different.

Carbohydrate value is a calculation by difference (Total carbohydrate—Crude fiber).

### Incremental area under the curve

The capillary blood glucose responses to reference and test foods from three separate GI tests of this study are summarized in Table 5. Glucose caused rapid and very high increase in the glycemic response of the volunteers. It picked to 6.78 mmol/L after 30 min of ingestion of the food and decreased steadily to 4.69 mmol/L at 120 min, a trend that was also observed for whole maize and grits flour porridges. As shown in Table 5, there were no significant differences in the fasting (at 0 min) blood glucose values for the 3 days of GI testing. However, after 15 min of post ingestion significant differences were observed in blood glucose responses between porridges prepared from grits and fermented flours.

**Table 5:** Blood glucose responses to thick porridges made from whole maize flour, dehulled, degermed maize flour, and maize flour made from fermented dehulled and degermed maize grits (mmol/L, *n* = 11)

Time (minutes)	Whole maize flour	Dehulled, degermed maize flour	Maize flour made from fermented dehulled and degermed maize grits	*P*‐value
0	4.10 ± 0.56	4.10 ± 0.79	3.80 ± 0.61	0.477
15	4.89 ± 0.75^ab^	5.24 ± 0.80^b^	4.40 ± 0.65^a^	0.040
30	6.06 ± 0.63^ab^	6.54 ± 0.71^b^	5.47 ± 0.95^a^	0.012
45	6.35 ± 0.65	6.66 ± 1.16	5.66 ± 1.11	0.069
60	6.14 ± 0.72^b^	6.36 ± 0.76^b^	5.01 ± 0.78^a^	<0.001
90	5.51 ± 0.93^b^	5.56 ± 0.95^b^	4.54 ± 0.85^a^	0.020
120	5.37 ± 0.72^b^	5.36 ± 0.91^b^	4.38 ± 0.83^a^	0.011

Values are means of 11 subjects, means with different superscripts in the same row are significantly different (*P *<* *0.05).

### GI of the test foods

As shown in Table 6, there were variations in GI responses across individuals and test foods. Observed differences ranged from 46.13–157.13 for whole maize flour porridge, 73.22–177 for porridge from grits flour, and 24.0–133.88 for fermented porridge. Individual GI responses for the three porridges were also different. Large differences between highest and lowest GI responses were observed for subjects number 2 (108), 11 (101.62), 3 (89.25), and 7 (85.35). Average values calculated showed that fermented porridge had the lowest GI of 65.49 followed by whole maize (94.06) and grits (109.64) flour porridges, respectively.

**Table 6:** Incremental area under the curve (iAUC) and glycemic indices of reference and test foods

Subject	Glucose	Whole maize flour	Dehulled, degermed maize flour	Maize flour made from fermented dehulled and degermed maize grits (Differences)	*P*‐value
1	62.56	99.38	73.22	54.75 (44.63)	
2	88.88	157.13	133.88	49.13 (108)	
3	130.13	69.38	118.88	29.63 (89.25)	
4	85.75	70.13	85.13	24.00 (61.13)	
5	85.95	73.50	77.22	58.00 (19.22)	
6	73.75	139.50	120.38	119.25 (20.25)	
7	120.63	90.00	129.00	43.65 (85.35)	
8	77.38	46.13	99.38	39.03 (60.35)	
9	125.25	109.13	82.50	93.75 (26.63)	
10	87.60	72.38	109.50	133.88 (61.5)	
11	86.72	108.00	177.00	75.38 (101.62)	
Mean iAUC	93.14 ± 22.16	94.06 ± 32.99^ab^	109.64 ± 30.86^b^	65.49 ± 36.18^a^	0.014
GI values		106.72 ± 47.83	121.97 ± 38.99	74.90 ± 46.22	0.055

Values for iAUC are means of 11 subjects and means with different superscripts in the same row are significantly different (*P *<* *0.05).

## Discussion

Changes in lifestyle and diet have contributed to an increased prevalence of diabetes in many low‐ and middle‐income countries, including Malawi, where the burden of infectious diseases, such as HIV/AIDS and TB, is already high. This dual burden of disease is a serious and growing challenge for health systems. The aim of this study was to investigate glycemic indices (GI) of staple stiff porridges, locally known as *nsima*, to identify areas for improvement in consumer diets. A rigorous review of the extant literature reveals that in Malawi only Chilenga (2012) and Mpulula (2013) have conducted scholarly research on processing methods of selected maize flour products and their impact on physiologic and sensory characteristics. Our study is the first of its kind in this regard and has important implications for nutritionists and healthcare practitioners.

Analysis of our descriptive data showed that GI responses varied across subjects and test foods. More so, 11 healthy subjects engaged were, at the very least, not aware of their blood glucose responses to staple foods they eat daily neither did they know the GI concept nor understand how it relates to diabetes. The problem is aggravated by lack of resources, poor health infrastructure, and inadequate laboratory facilities typical of developing countries and, likely, people die from the disease without ever being diagnosed. Latest statistics show that six in every 100 people (5.6%) in Malawi are diabetes sufferers and 3.9% of the cases remain undiagnosed (Msyamboza et al. 2014).

In this study, fermented porridge has shown to have the lowest GI of 74.90 (Table 6). We recommend fermented flour to be promoted for the preparation of various maize‐based foods meant for the diabetics in the country. This flour has also shown to have most preferred sensory characteristics for the majority of local consumers (Chilenga 2012). The other two types of *nsima* tested had higher GI values and the potential to raise blood glucose in the body. Table 6 shows that a 50 g portion of *nsima* made from whole maize flour had a GI of 106.72. Paradoxically, this was the test food that showed to have the highest amount of protein, fat and dietary fiber (Table 4). These results suggest that *nsima* made from whole maize flour may also be a good choice for the general population considering the problem of malnutrition in the country and since local side dishes used have low GI, such as legumes, vegetables, and sauces. In every 100 Malawian children less than 5 years of age 48 are stunted, 13 underweight and five wasted (National Statistical Office, 2010). Foods rich in both macro and micronutrients are needed to combat the problem.

Previous studies undertaken elsewhere in Africa show mixed results for GI of maize flour products. Foster‐Powell et al. (2002) have summarized figures ranging from 71 to 109 for studies conducted in South Africa and Kenya. In Nigeria, values reported range from 26.6 to 54.83 (Fasanmade and Anyakudo 2007) and 86.8–92.3 in the study by Panlasigui et al. (2010). These variations can be explained at least in part by discrepancies in specific test foods, traditional food preparation methods used as well as differences in sample sizes used, which ranged from 8 to 50, and characteristics of study subjects (healthy vs type 2 diabetes mellitus patients).

We found that glycemic index and glycemic load patterns (calculated as GL = GI/100 × CHO grams per serving) were consistent. Glycemic load measures the degree of glycemic response and insulin demand produced by a specific amount of a specific food (FAO/WHO, 1998). While GI ranks carbohydrates based on their immediate blood glucose response, GL reflects both the quality and quantity of dietary carbohydrates. In addition to this, GL helps predict blood glucose response to specific amount of specific carbohydrate food. Glycemic loads for whole maize, grits and fermented grits flour porridges were 47.03, 54.82, and 32.75 respectively. We predict that porridges cooked using the current Malawian maize flours and recipes would continue to raise blood sugar levels in the long‐term considering high‐GL figures recorded (defined as ≥20).

There is no easy way to change people's diets and food habits developed through experiences over the life course (Furst et al. 1996; Rozin 1996; Clarke 1998; EUFIC, 2005). According to the Health Belief Model (HBM) proposed by Rosenstock (1966) and modified later by Becker (1974), people need some kind of cue, such as being personally threatened by a disease, to take action with respect to changing dietary behavior. Taken together, considering that *nsima* is the main meal in Malawi interventions that stress small diet changes and consumer education are likely to succeed. An example of how to achieve this change is replacing maize with another staple, such as cassava, rice, and potatoes, at one meal to start with and then slowly increasing the amount of these staples until they are fully incorporated into food habits.

The study is not without limitations. Our data have limitations similar to other studies that measured GI based on 50 g available carbohydrate of single food as a reference amount for GI testing. In real life situations, people eat larger portion sizes of food and in this regard *nsima* is always eaten with sauces, including meat, legumes, fish, and vegetables. Classification of GI responses using relative glycemic effect (RGE) method, which is based on total serving size, could help minimize the bias in this regard. Moreover, a day before the test each subject ought to consume a meal of choice and repeat that meal before each test. This meal pattern was difficult to follow because subjects were not under controlled conditions. Different types of food and nutrients (simple sugars, carbohydrates, and fats) have different chemical compositions and should vary in terms of the amount of energy released (amount of heat per gram of food burned) during the calorie test. Unfortunately, the test was not conducted in a bid to avoid making the study too broad for researchers.

## Conclusion

In conclusion, notwithstanding the aforesaid limitations this study has implications for future research on diet and prevention of diabetes mellitus and related diseases in Malawi. We allude that diet cannot by itself suffice to curb the current burden of noncommunicable diseases in light of their complexity and healthcare infrastructure constraints in the country. Interventions need to be multidisplinary and wide reaching in approach, encompassing issues of consumer education, policy framework, and enabling environment for stakeholders to be effective in this regard.

## Conflict of Interest

Authors have no conflict of interest to disclose.

## References

[fsn3293-bib-0001] AOAC . 2002 Official methods of analysis of association of official analytical chemist, 17th edn. Association of Official analytical Chemist, Arlington, VA, USA.

[fsn3293-bib-0002] Becker, M. H. 1974 The health belief model and sick role behavior. Health Educat. Monog. 2:409–419.

[fsn3293-bib-0003] Benton, D. , M. P. Ruffin , T. Lassel , S. Nabb , M. Messaoudi , S. Vinoy , et al. 2003 The delivery rate of dietary carbohydrates affects cognitive performance in both rats and humans. Psychopharmacology (Berlin) 166:86–90. 12488949 10.1007/s00213-002-1334-5

[fsn3293-bib-0004] Brand‐Miller, J. M. D. , K. M. Foster‐Powell , and J. McMillan‐Price . 2014 The low GI diet revolution: the definitive science‐based weight loss plan. Da Capo Press, Boston, MA.

[fsn3293-bib-0005] Brouns, F. I. , K. N. Bjorck , A. L. Frayn , V. Gibbs , G. Slama Lang , and T. M. S. M. Wolever . 2005 Glycaemic index methodology. Nutr. Res. Rev. 18:145–171. 19079901 10.1079/NRR2005100

[fsn3293-bib-0006] Chilenga, C . 2012 Effect of variety and processing methods on physiological characteristics of maize flour and sensory properties of maize nsima. Master of Science Thesis, University of Malawi, Bunda College of Agriculture, Lilongwe. Malawi.

[fsn3293-bib-0007] Clarke, J. E. 1998 Taste and flavour: their importance in food choice and acceptance. Proceed. Nut. Soc. 57:639–643. 10.1079/pns19980093 10096128

[fsn3293-bib-0008] EUFIC [The European Food Information Council] . 2005 The determinants of food choice. http://www.eufic.org/article/en/expid/review-food-choice/ (accessed 20 February 2015).

[fsn3293-bib-0009] Fasanmade, A. A. , and M. M. C. Anyakudo . 2007 Glycaemic indices of selected Nigerian flour meal products in male type 2 diabetes subjects. Diabetologia Croatica 36:33–38.

[fsn3293-bib-0010] Fatema, K. , C. B. Mikkelsen , F. Rahman , N. Sumi , and L. Ali . 2013 Glycemic and insulinemic responses to isis cookies and Danish Traditional Cookies in Healthy Subjects. J. Food Nut. Disord. 2:1–5.

[fsn3293-bib-0011] Food and Agriculture Organization of the United Nations(FAO) . 1992 Maize in human nutrition, introduction. FAO; Food and Nutrition Series, 25. Http://www.fao.org/docrep/t0395e/T0395E00.htm (accessed 1 August 2013).

[fsn3293-bib-0012] Food and Agriculture Organization of the United Nations (FAO)/World Health Organization (WHO) . 1998 Expert consultation. Carbohydrates in human nutrition: report of a joint FAO/WHO Expert Consultation, Rome, 14–18 April, 1997. Rome: Food and Agriculture Organization, (FAO Food and Nutrition paper 66). http://www.who.int/nutrition/publications/nutrientrequirements/ scientific_update_carbohydrates/en/[Accessed on 12 August 2013].

[fsn3293-bib-0013] Foster‐Powell, K. , S. H. Holt , and J. C. Brand‐Miller . 2002 International table of glycaemic index and glycaemic load values. Am. J. Clin. Nutr. 76:5–56. 12081815 10.1093/ajcn/76.1.5

[fsn3293-bib-0014] Furst, T. , M. Connors , C. A. Bisogni , J. Sobal , and L. Winter Falk . 1996 Food choice: a conceptual model of the process. Appetite 26:247–266. 8800481 10.1006/appe.1996.0019

[fsn3293-bib-0015] Government of Malawi . 2012 Health sector strategic plan 2011–2016. Likuni Press and Publishing House, Lilongwe, Malawi.

[fsn3293-bib-0017] Kelly, S. A. M , G. Frost , V. Whittaker , and C. D. Summerbell . 2004 Low glycaemic index diets for coronary heart disease. Cochrane Database of Systematic Reviews. 4: 1–66. 10.1002/14651858.CD004467.pub2 15495112

[fsn3293-bib-0018] Mahgoub, S. O. , M. Sabone , and J. Jackson . 2013 Glycaemic index of selected staple carbohydrate‐rich foods commonly consumed in Botswana. South Afr. J. Clin. Nut. 26: 182–187.

[fsn3293-bib-0019] Matumba, L. , M. Monjerezi , E. Chirwa , D. Lakudzala , and P. Mumba . 2009 Natural occurrence of AFB1 in maize and effect of traditional maize flour production on AFB1 reduction in Malawi. Afr. J. Food Sci. 3:413–425.

[fsn3293-bib-0020] Meyer, K. A. , L. H. Kushi , D. R. Jacobs , J. Slavin , T. A. Sellers , and A. R. Folsom . 2000 Carbohydrates, dietary fibre and incident type 2 diabetes in older women. Am. J. Clin. Nutr. 71:921–930. 10731498 10.1093/ajcn/71.4.921

[fsn3293-bib-0021] Mpulula, O . 2013 Effect of home‐based maize processing methods and variety on apparent iron and zinc bio‐availability. M.Sc. thesis. University of Malawi, Bunda College of Agriculture. Lilongwe. Malawi.

[fsn3293-bib-0022] Msyamboza, K. P. , C. J. Mvula , and D. Kathyola . 2014 Prevalence and correlates of diabetes mellitus in Malawi: population‐based national NCD STEPS survey. BMC End. Disord. 14:41. 10.1186/1472-6823-14-41 PMC4018960 24884894

[fsn3293-bib-0023] National Statistical Office (NSO) . 2010 Malawi demographic and health survey. NSO, Zomba, Malawi.

[fsn3293-bib-0025] Panlasigui, L. N. , C. L. T. Bayaga , E. B. Barrios , and K. L. Cochon . 2010 Glycaemic response to quality protein maize grits. J. Nut. Metab. Pp 1‐6 http://dx.doi.org/10.1155/2010/697842. 10.1155/2010/697842 PMC2938446 20862364

[fsn3293-bib-0026] Rizkalla, S. W. , L. Taghrid , M. Laromiguiere , D. Huet , J. Boillot , A. Rigoir , et al. 2004 Improved plasma glucose control, whole‐body glucose utilization, and lipid profile on low‐glycemic index diet in type 2 diabetic men: a randomized controlled trial. Diabetes Care 27:1866–1872. 15277409 10.2337/diacare.27.8.1866

[fsn3293-bib-0100] Rosenstock, I. M. 1966 Why people use health services. Milbank Memorial Fund Quarterly. 83:1–32. 5967464

[fsn3293-bib-0027] Rozin, P. 1996 Sociocultural influences on human food selection Pp. 233–263 *in* Capaldi E. D., ed. Why we eat what we eat: the psychology of eating. American Psychological Association, Washington, DC.

[fsn3293-bib-0101] Wolever, T. M. , and C. Mehling . 2003 Long‐term effect of varying the source or amount of dietary carbohydrate on postprandial plasma glucose, insulin, triacylglycerol, and free fatty acid concentrations in subjects with impaired glucose tolerance Am. J. Clin. Nutr: 77: 612–621. 10.1093/ajcn/77.3.612 12600851

